# Quercetin and Cancer Chemoprevention

**DOI:** 10.1093/ecam/neq053

**Published:** 2011-04-14

**Authors:** Lara Gibellini, Marcello Pinti, Milena Nasi, Jonas P. Montagna, Sara De Biasi, Erika Roat, Linda Bertoncelli, Edwin L. Cooper, Andrea Cossarizza

**Affiliations:** ^1^Department of Biomedical Sciences, University of Modena and Reggio Emilia School of Medicine, 41125 Modena, Italy; ^2^David Geffen School of Medicine, UCLA Medical Center (CHS), Los Angeles, CA, USA

## Abstract

Several molecules present in the diet, including flavonoids, can inhibit the growth of cancer cells with an ability to act as “chemopreventers”. Their cancer-preventive effects have been attributed to various mechanisms, including the induction of cell-cycle arrest and/or apoptosis as well as the antioxidant functions. The antioxidant activity of chemopreventers has recently received a great interest, essentially because oxidative stress participates in the initiation and progression of different pathological conditions, including cancer. Since antioxidants are capable of preventing oxidative damage, the wide use of natural food-derived antioxidants is receiving greater attention as potential anti-carcinogens. Among flavonoids, quercetin (Qu) is considered an excellent free-radical scavenging antioxidant, even if such an activity strongly depends on the intracellular availability of reduced glutathione. Apart from antioxidant activity, Qu also exerts a direct, pro-apoptotic effect in tumor cells, and can indeed block the growth of several human cancer cell lines at different phases of the cell cycle. Both these effects have been documented in a wide variety of cellular models as well as in animal models. The high toxicity exerted by Qu on cancer cells perfectly matches with the almost total absence of any damages for normal, non-transformed cells. In this review we discuss the molecular mechanisms that are based on the biological effects of Qu, and their relevance for human health.

## 1. Introduction

### 1.1. Chemoprevention and Diet

The field of cancer chemoprevention, defined as the long-term intervention with natural or synthetic molecules to prevent, inhibit or reverse carcinogenesis, is gaining increasing importance, especially at a time when the use of complementary and alternative medicine (CAM) and natural health products is consistently increasing [1]. At present, the use of CAM in oncology represents a challenging area of interest since remarkable scientific evidences suggest that natural dietary factors can inhibit the process of carcinogenesis and can effectively influence the risk of cancer in humans. The idea of cancer chemoprevention arises from both statistical and epidemiological data showing that a modification of the lifestyle is associated with lower incidence of certain types of cancers (i.e., colorectal, stomach, lung and esophageal cancers) and that, in particular, such modifications include a vegetable- and fruit-rich diet [2].

Accumulating evidences from observational and prospective studies indicate that dietary components may substantially alter the natural history of carcinogenesis, and that an inverse correlation between a high consumption of fruits and vegetables and the incidence of some cancers does exist [3, 4]. Indeed, a high consumption of fruits and vegetables was associated with a reduced risk of intestinal cancer, especially of colon cancer, in former smokers or in no-smokers, whereas no preventive effect was found in current smokers [5]. Consumption of fruits, particularly citrus fruit, as well as vegetables, likely correlates with decreased esophageal cancer risk [6, 7]. Green leafy vegetables, rather than fruit, might also have a protective effect against lung cancer [8, 9]. High intake of cruciferous vegetables may be associated with reduced risk of aggressive prostate cancer [10]. With regard to renal cancer, although the results of case-control studies are not fully consistent and often contradictory, analyses performed on very large number of patients have found an inverse association for the intake of total fruit [11], total vegetables [12], and some subgroups of vegetables such as cruciferous vegetables, dark green vegetables and yellow-orange vegetables [13]. Conversely, positive effects of fruit and vegetable consumption, combined or separately, were not found as far as the risk of developing bladder or ovary cancer was concerned [14].

### 1.2. Natural Chemopreventers

The majority of the case-control studies focused on the use of fruits and vegetables because these items, which include soybean, ginger, onion, cabbage, cauliflower, turmeric, are the basis of most diets throughout the world, and represent important sources of potentially non-toxic molecules (dietary phytochemicals). These molecules can exert a cancer-preventive effect and therefore are termed as “chemopreventers" [15]. Among them, the most studied are curcumin, quercetin (Qu), resveratrol, luteolin, genistein, (−)-epigallocatechin-3-gallate (EGCG), lycopene and, in general, flavonoids and polyphenols [16–19]. A majority of studies have analyzed the biological properties (e.g., antioxidant, antimicrobial, antiproliferative, pro- or anti-apoptotic) of the aforementioned molecules that are present, along with other compounds, in aqueous extracts from plants [20–25].

A relevant interest is present on the mechanism(s) of action of chemopreventers, especially concerning the identification of molecular and cellular targets of CAM compounds, and the molecular basis of their cancer-preventive action. At the biochemical level, chemopreventers usually act as modulators of signal transduction pathways that are involved in almost all biological processes: cell proliferation, apoptosis, cell migration, cell differentiation, oxidative balance and inflammation [26–29]. Chemopreventers often have preferentially an antioxidant activity; however, they are also able to exert anti-proliferation and anti-inflammation actions. Indeed, they can directly modulate several proteins that are involved in cell cycle and cellular homeostasis and whose deregulation can play a role in carcinogenesis, such as p53, p73, p21, Bax, Bcl-2, COX-2, NF-kB, catalase, glutathione (GSH)-peroxidase [4, 30, 31]. The antioxidant activity of chemopreventers is nowadays gaining more importance because of the observations, both *in vitro* and *in vivo*, that the deregulation of free-radical homeostasis can be involved in carcinogenesis [32].

## 2. Carcinogenesis and Reactive Oxygen Species

### 2.1. Reactive Oxygen Species Induced Carcinogenesis in Animal Models

The evidence of a strong association between the production of free radicals and carcinogenesis mainly derives from *in vitro* studies showing that some pro-oxidant chemicals promote tumors in several animal models, whereas primary endogenous antioxidant enzymes could interfere with tumor promotion. For instance, 1,2-dimethylhydrazine induces colon carcinogenesis in rats [33, 34], whereas benzoylperoxide promotes papillomas and carcinomas after 7,12-dimethylbenz[a]anthracene initiation in mice [35]. In rat liver epithelial cells treated with *N*-methyl-*N*′-nitro-*N*-nitrosoguanidine, hydrogen peroxide exerts a tumor promoting activity [36]. Conversely, the overexpression of manganese superoxide dismutase (MnSOD) reduces tumor incidence in a multistage skin carcinogenesis mouse model [37]. A combined deficiency in two mitochondrial-localized antioxidant enzymes, MnSOD and GSH peroxidase-1 (Gpx-1), determines an increased incidence of neoplasms in mice [38].

### 2.2. Reactive Oxygen Species Induced Carcinogenesis in Human Cells

The involvement of reactive oxygen species (ROS) in tumor progression has also been demonstrated in human cells. NADPH oxidase 1 (Nox1), an enzyme that produces superoxide (which is in turn dismuted to hydrogen peroxide), is overexpressed in colon and prostate cancer cell lines [39, 40], while its downregulation reverses tumor growth [41]. Decreased levels of MnSOD and rapid cell doubling time have been reported in human pancreatic cancer cell lines at various levels of differentiation [42].

These studies are consistent with the observation that a significant shift of cellular oxidative balance could lead to tumor promotion or progression, as ROS are involved in damaging of DNA as well as in mitogenic signaling [43–45]. Endogenous ROS are generally supposed to cause DNA damage [46], through the production of oxidized bases and DNA strand breaks, and to be a relevant factor contributing to chromosome instability and accumulation of mutations and deletions, finally leading to cancer [47]. About 1% of oxygen consumption results in the production of ROS [46], thus implying that, in every cell, ROS can damage ∼20 000 DNA bases per day [48]. Cells have evolved several antioxidant defenses, including repair and detoxifying enzymes, and small scavenger molecules, such as GSH. Nevertheless, the presence of these intracellular protective systems is not sufficient to ensure an adequate and complete removal of oxidative damages.

Apart from the direct action on DNA, it has to be noted that ROS act as secondary messengers in several pathways, which can potentially promote carcinogenic processes, including resistance to apoptosis, increase in cell proliferation and production of metastasis [32]. Indeed, ROS have been involved in the transcriptional activation of several proto-oncogenes, such as c-FOS, c-JUN and c-MYC. In human hepatoma cells, ROS modulate the expression of c-FOS and c-JUN through PKB pathway [49]. Furthermore, p66Shc, which is involved in the regulation of ROS signaling, is responsible for androgenic proliferation signals through ROS production in prostate cancer cells that are positive to androgen receptor [50]. Finally, in anaplastic large cell lymphomas, the use of nordihydroguaiaretic acid, which is an inhibitor of lipoxygenase, results in the inhibition of several pathways which are involved in antiapoptotic and pro-mitogenic functions [51].

## 3. Flavonoids and Their Possible Role in Cancer Chemoprevention

### 3.1. Flavonoids in the Diet

Among chemopreventers, one of the most studied group of antioxidant compounds are flavonoids. Flavonoids are a large heterogeneous group of benzo-*γ*-pyrone derivatives that share a common carbon skeleton of dyphenylpropanes [52] and can be divided into six different classes, namely flavonols, flavones, flavanones, flavanols, isoflavones and anthocyanidins, according to their molecular structure [53].

Flavonoids are largely present in fruits, vegetables, aromatic plants, medical herbs, tea and red wine [54]. It is extremely difficult to estimate the daily human intake of flavonoids, especially because of the lack of standardized analytical methods [55]. However, the average daily intake of the most abundant flavonoids, catechins, is ∼100 mg [56]. Similar to daily intake, it is also quite complex to assess and quantify the bioavailability of flavonoids [57]. Nevertheless, metabolized forms of flavonoids present in blood significantly differ from the native compounds, and plasma concentration of total metabolites can have a range 0–4 *μ*mol L^−1^ with an intake of 50 mg of aglycone, which is the non-sugar compound left after partial metabolization of the original flavonoid [58].

### 3.2. Cancer Chemoprevention by Flavonoids: Molecular Mechanisms

Results from cell culture and animal models reveal that flavonoids exert positive preventive effects in carcinogenesis and neurodegenerative disorders essentially because of their antioxidant activity, their capacity to affect the expression of several detoxifying enzymes [59], and their ability to modulate protein signaling cascades [60]. Flavonoids can interfere with specific stages of the carcinogenic process, and can inhibit cell proliferation and induce apoptosis in several types of cancer cells.

EGCG is one of the most intensively studied flavonoids as it is the major polyphenolic component of green tea. EGCG inhibits cell proliferation and induces apoptosis in several human tumor cell lines, including CaSki and HeLa cervical cells [61], Hep-2 cells [62], laryngeal squamous carcinoma cells [63], SW780 and TCCSUP bladder urothelial cells [64], melanoma cells [65], adrenal NCI-H295 cancer cells [66] and A549 lung cancer cells [67]. The mechanisms by which apoptosis is triggered differ depending on the cell line and include via death receptor, or via mitochondrial and endoplasmic reticulum-dependent pathways.

The cancer-preventive properties of flavonoids can be attributed to their capacity of quenching ROS, reactive nitrogen species (RNS) and other radicals. Tea catechins, especially EGCG, react with superoxide radical, hydroxyl radical, peroxyl radical and peroxynitrite [68]. Resveratrol, present in red wine, grapes and peanuts, is a scavenger of superoxide and peroxynitrite radicals [69], and genistein, mainly derived from soy, can scavenge exogenous or endogenous hydrogen peroxide in cell models [70]. Moreover, flavonoids exert their protective antioxidant effect not only by quenching ROS, but also by modulating the activity of several detoxifying enzymes, including lipoxygenase, cycloxygenase, inducible nitric oxide synthase, monoxygenase, xanthine oxidase and NADH oxidase [71–75]. Among enzymes that are inhibited by flavonoids, thioredoxin reductases have to be quoted, as they are involved in cellular redox control, and are overexpressed in different aggressive tumors [76].

Growing evidences suggest that flavonoids (in particular, resveratrol and quercetin) may contribute to chromatin remodeling and thus interfere with epigenetic alterations that are important in cancer progression. Chromatin is remodeled by chemical modifications of DNA and histones, such as DNA methylation and multiple histone modifications, such as methylation, phosphorylation, acetylation, sumoylation and ubiquitination; for example, resveratrol activates sirtuin (SIRT)-1, a member of histone deacetylase (HDAC) family, which plays key roles in cell survival and apoptosis [77]. The network of SIRT1-modulated signals is wide and complex, and involves SIRT1 direct interactions with several proteins involved in cell survival (p53, bax, E2F1, FOXO3, Dif1), DNA repair (WRN, Ku70, RAD51) and cell cycle/apoptosis (*β*-catenin, survivin, NF*κ*B) [78]. The activation of SIRT1 by resveratrol induces the formation of SIRT1-p300 complexes, causing the inactivation of p300 acetyltransferase and a reduction in the acetylation of both *β*-catenin and NF*κ*B-p65. The main consequence of this phenomenon is the downregulation of the multidrug resistance (MDR)-1 and Bcl-xL genes with the subsequent stimulation of cell death, as well as the reduction of chemoresistance in breast tumor cells [79].

Several catechol-containing dietary polyphenols are capable of modulating DNA methylation. Among them, EGCG is a potent and efficacious *in vitro* inhibitor of DNA methyltransferase (DNMT)-1, whereas Qu can demethylate the p16INK4a gene promoter, whose hypermethylation is present in human colon cancer cells [80]. Qu also activates histone deacetylase enzymatic activity, thus reducing the acetylation of histone H3 in human prostate cancer cells. The deacetylation of H3 could be responsible for the inhibition of survivin expression, and for the subsequent sensitization to TRAIL-induced apoptosis [81].

### 3.3. Cancer Chemoprevention by Flavonoids in Human

Regarding flavonoids as chemopreventers in humans, contrasting results have been reported, and indeed some studies showed an inverse correlation between the intake of total dietary flavonoids and the risk of cancer [82–85], whereas others did not evidence any association [86]. Furthermore, the importance of risk factors such as smoke has to be taken into account, since in different groups of patients, a limited evidence for a preventive effect of flavonoids on the development of pancreatic cancer has been reported. For example, no association between flavonoid intake and pancreatic cancer risk was found in male current smokers [87]. The Multiethnic Cohort Study, on the contrary, reported that the intake of total flavonols was associated with a reduced pancreatic cancer risk among current smokers, but not in never or former smokers [88]. Lack of preventive effect was described in the case of ovarian cancer [89]. Conversely, several other epidemiological studies confirmed the protective role of a high flavonoid intake against colorectal [90, 91] and lung cancers [92]. Interestingly, although natural chemopreventers have undergone extensive mechanistic investigation at the molecular and cellular level, their preclinical efficacy needs to be further explored, and clinical trials have only recently started to investigate the potential preventive role of these compounds [93].

## 4. Qu and Its Molecular Role in Cancer Chemoprevention

### 4.1. The Importance of the Diet

Qu (3,3′,4′,5,7-pentahydroxyflavone) is an important dietary flavonoid, present in different vegetables, fruits, seeds, nuts, tea and red wine [94–96]. The average daily intake of Qu can reach 30 mg in most Western countries [97], and its bioavailability depends on the metabolic form present in the food. Indeed, Qu obtained from plant source is in the form of Qu-glucose conjugates (Qu glucosides), which are absorbed in the apical membrane of the enterocytes. Once absorbed, Qu glucosides are hydrolyzed to generate Qu aglycone which is further metabolized to the methylated, sulfonylated and glucuronidated forms by the enterocytic transferases [98]. Qu metabolites are then transported first to the intestinal lumen [98], and then to the liver, where other conjugation reactions take place to form Qu-3-glucuronide and Qu-3′-sulfate, which are the major Qu-derived circulating compounds in human plasma [99, 100]. According to recent studies on Qu bioavailability [58], when Qu is absorbed in the form of Qu glucosides, the peak plasma concentration ranges from 3.5 to 5.0 *μ*mol L^−1^. In the unconjugated form, Qu absorption is less efficient, and peak plasma concentration is <0.33 *μ*mol L^−1^.

The study of Qu as potential chemopreventer is assuming increasing importance considering its involvement in the suppression of many tumor-related processes including oxidative stress, apoptosis, proliferation and metastasis. Qu has also received greater attention as pro-apoptotic flavonoid with a specific and almost exclusive activity on tumor cell lines rather than normal, non-transformed cells [101].

### 4.2. Two Faces of the Same Molecule: Anti-Oxidant and Pro-Oxidant Properties

Qu is considered an excellent free-radical scavenging antioxidant owing to the high number of hydroxyl groups and conjugated *π* orbitals by which Qu can donate electrons or hydrogen, and scavenge H_2_O_2_ and superoxide anion (•O_2_^−^) [102]. The reaction of Qu with •O_2_^−^ leads to the generation of the semiquinone radical and H_2_O_2_ [103]. Qu also reacts with H_2_O_2_ in the presence of peroxidases, and thus it decreases H_2_O_2_ levels and protects cells against H_2_O_2_ damage; nevertheless, during the same process potentially harmful reactive oxidation products are also formed. The first oxidation product of Qu is a semiquinone radical [103]. This radical is unstable and rapidly undergoes a second oxidation reaction that produces another quinone (Qu-quinone, QQ) [103]. Since QQ can react with proteins, lipids and DNA, it is responsible for protein and DNA damage as well as lipid peroxidation. As far as DNA is concerned, QQ can mediate DNA strand breaks and can induce the oxidation of 2′-deoxyguanosine to form 8-hydroxy-2′-deoxyguanosine [104].

**Figure 1:**
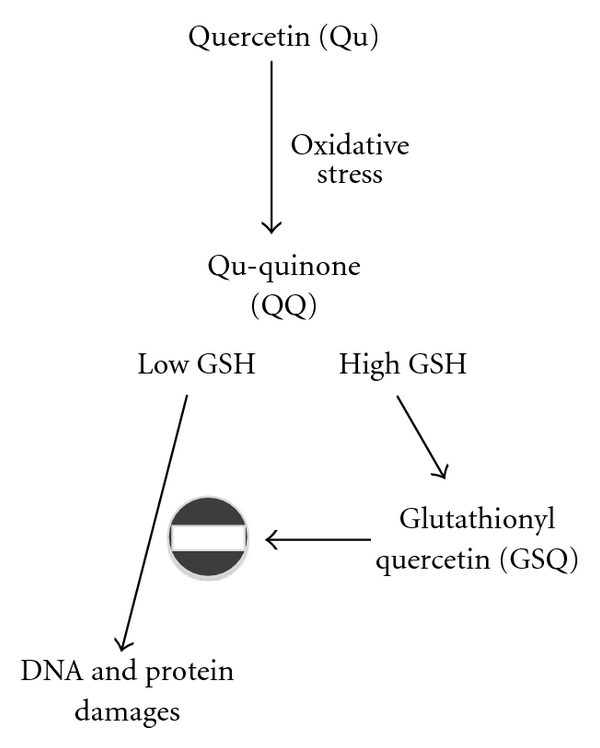
Antioxidant and pro-oxidant effects of Qu in the presence of low and high levels of reduced GSH. The antioxidant and pro-oxidant effects of Qu strongly depend upon the availability of intracellular reduced GSH. During an oxidative stress, in the presence of peroxidases, Qu reacts with H_2_O_2_ to form a semiquinone radical that is rapidly oxidized to QQ. QQ has a pro-oxidant effect; its high reactivity towards protein thiols and DNA leads to cell damage and cytotoxicity. QQ is also highly reactive towards thiols, and preferentially reacts with GSH to form relatively stable protein-oxidized Qu adducts such as 6-GSQ and 8-GSQ. The reversibility of this reaction allows the continuous dissociation of GSQ into GSH and QQ. In the presence of high GSH concentrations, QQ reacts with GSH to form GSQ again, and QQ cannot exert its cytotoxic effects, whereas when low levels of GSH are present, QQ reacts with protein thiols, thus leading to cellular damage.

Biochemically, QQ is highly reactive towards thiols and preferentially reacts with reduced GSH to form relatively stable GSH-oxidized Qu, that is, 6-glutathionyl-Qu (GSQ) and 8-GSQ [105]. Along with 6-GSQ and 8-GSQ, the generation of 2′-GSQ has also been characterized in dermal fibroblasts [106]. The reaction leading to the formation of GSQ is reversible and glutathionyl-Qu adducts can be continuously dissociated into QQ and GSH [107]. As a result, in the presence of high GSH concentrations, oxidized Qu reacts with GSH to form GSQ again, and the reversibility of the reaction ensures the protection against QQ toxicity. In the presence of low GSH content, oxidized Qu reacts with protein thiols, exerting a toxic effect within cells [107, 108]. Similarly, long exposure to Qu along with high Qu concentration, causes a reduction in GSH content, suggesting the inability of Qu to cope with ROS for that period. As a consequence, the pro-oxidant effect of Qu could prevail over the antioxidant effect and result in cell death by damaging cellular compartments [109, 110]. As shown in Figure 1, when high levels of GSH are present, Qu-derived semiquinone and quinoidal products are constantly reduced, thus limiting Qu cytotoxicity and enabling Qu to act as antioxidant rather than as pro-oxidant [111]. The antioxidant capability of Qu strongly depends on the intracellular availability of GSH, since, in Qu-treated cells, alterations typical of apoptosis appear when intracellular GSH is completely depleted. Indeed, in different cellular models low concentrations of Qu induce cell proliferation and increase the antioxidant capacity of the cells, whereas higher concentrations of Qu decrease antioxidant capacity and thiol content, ultimately causing cell death [112].

### 4.3. Cell Cycle as a Possible Target

**Figure 2:**
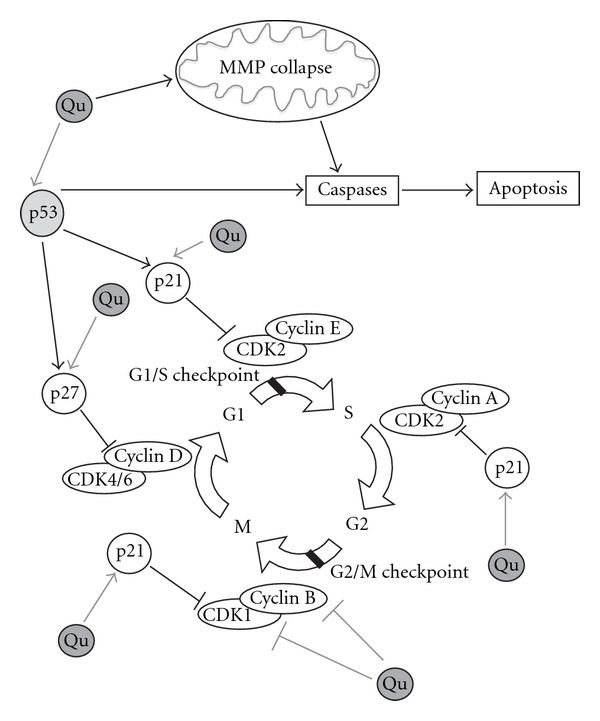
Effects of Qu on cell cycle. Qu is able to regulate cell cycle by directly binding several molecular targets and, depending on the cell type and tumor origin, it blocks the cell cycle at G2/M or at the G1/S transition. At the G1/S transition, Qu blocks cell-cycle progression through the up-regulation of p21 and p27 and p53. p21 exerts an inhibitory activity on several CDKs. In particular, p21 inhibits CDK2-cyclin E, with the consequent inhibition of CDK2-dependent phosphorylation of pRb and the sequestration of E2F1, thus inhibiting gene transcription induced by E2F1 and progression into and through S phase. p21 also inhibits CDK2-cyclin A and CDK1-cyclin B, which are essential for progression through S phase and G2, respectively. p27 exerts several effects on cell cycle, but only under certain conditions it can inhibit the complexes CDK4-cyclin D and CDK6-cyclin D. The tumor suppressor p53, once activated, can induce several different cellular responses, including growth arrest and apoptosis. Growth arrest is essentially elicited through the up-regulation of the genes that encode for inhibitors of cell-cycle progression, including p21 and p27. In different cellular models, Qu stabilizes p53 both at mRNA and protein levels. Apart from blocking cell growth through the direct action on key modulators of cell cycle, Qu is able to induce apoptosis via mitochondrial pathway: indeed, Qu can disrupt MMP, which in turn provokes the release of cytochrome *c* in the cytoplasm, a phenomenon that activates multiple caspases, such as caspase-3 and -7.

Apart from scavenging ROS, another important effect of Qu is to regulate cell cycle by modulating several molecular targets, including p21, cyclin B, p27, cyclin-dependent kinases and topoisomerase II, even if the mechanisms involved have not been elucidated yet. Depending on the cell type and tumor origin, Qu is able to block the cell cycle at G2/M or at the G1/S transition (Figure 2). In particular, Qu causes G2/M arrest in human esophageal squamous cell carcinoma cell line through up-regulation of p73 and p21waf1 and subsequent down-regulation of cyclin B1, both at the mRNA and protein levels [113]. In human breast carcinoma cell lines such as SK-Br3, MDA-MB-453 and MDA-MB-231 cells, low doses of Qu inhibit proliferation. Cell-cycle arrest occurs at the G1 phase through the induction of p21 and through the concomitant decrease of phosphorylation of the retinoblastoma protein (pRb). In the same cell model, Qu downregulates the cyclin B1 and cyclin-dependent kinase (CDK) 1, which are essential in the progression to the G2/M phases of the cell cycle [114]. Similarly, in the human lung cancer cells NCI-H209, Qu glucuronides induce cell-cycle arrest at G2/M phase by increasing the expressions of proteins such as cyclin B, Cdc25c-ser-216-p and Wee1 [115]. A similar antiproliferative effect has also been observed both for highly or moderately aggressive prostate cancer cell lines, whereas no effect has been found for poorly aggressive prostate cancer cells [116]. In HepG2 human hepatoma cells, Qu blocks cell-cycle progression at the G1 phase, and exerts this effect through the increase of p21 and p27 and p53 [117]. Similar effects on the cell cycle have also been reported in SW872 cells [112].

Topoisomerase II (TopoII) is another potential and delicate target of Qu [118, 119]. Of note, the ability of Qu to directly poison TopoII through the stabilization of double strand breaks in the TopoII-DNA cleavage complexes could account for genetic rearrangements leading primary hematopoietic progenitor cells to develop mixed-lineage leukemia [120].

### 4.4. Direct Pro-Apoptotic Effects of Qu

Collectively, the pro-apoptotic effects of Qu may result from multiple pathways. First, in MDA-MB-231 cells, Qu treatment increases cytosolic Ca^2+^ levels and reduces the mitochondrial membrane potential (MMP), thus promoting activation of caspase-3, -8 and -9 [26]. The capability of Qu to induce apoptosis via mitochondrial pathway has been confirmed in U937 cell line [109, 121]. In these cells, Qu disrupts MMP [109, 121], which in turn provokes the release of cytochrome *c* in the cytoplasm [122], and subsequently activates multiple caspases, such as caspase-3 and -7 [123]. Second, Qu inhibits cell growth and apoptosis by down-regulating the transcriptional activity of *β*-catenin/Tcf signaling, with the consequent down-regulation of cyclin D1 and survivin [124, 125]. The Qu-induced regulation of apoptosis through the modulation of survivin has been demonstrated to have a controversial fashion in glioma cells as well as in lung carcinoma cell lines [126, 127]. Thus, while in glioma cells Qu exposure results in proteasomal degradation of survivin [127], according to another proposed model, Qu treatment raises cyclin B1 and p53 proteins that, in turn, increase survivin and p21 protein expression, thereby inhibiting apoptosis [126]. Third, Qu likely triggers apoptosis through the generation of ROS and the subsequent activation of AMPK*α*1 and ASK1 which is, in turn, accompanied by p38 activation and recruitment of caspases [63]. Fourth, the antiproliferative and pro-apoptotic effects could be related to the capability of Qu to directly bind tubulin, provoking the depolymerization of cellular microtubules [128]. Fifth, Qu is a potent enhancer of TNF-related apoptosis-inducing ligand (TRAIL)-induced apoptosis, through the induction of the expression of death receptor (DR)-5, a phenomenon that specifically occurs in prostate cancer cells [129]. The up-regulation of DR5, together with the down-regulation of c-FLIP (which is an inhibitor of caspase-8), are two mechanisms involved in Qu-induced recovery of TRAIL sensitivity, at least in hepatocellular carcinoma cells [130]. Of note, the enhancement of TRAIL-induced apoptosis by Qu also occurs through the inhibition of the expression of survivin in the ERK-MSK1 signal pathway [81].

Thus, the capability of Qu to induce apoptosis in cancer cells (via both the intrinsic and extrinsic pathways) undoubtedly renders this molecule an interesting tool in the oncology field.

### 4.5. Qu Influences p53 Activity

Several studies have investigated the role of p53 in the antiproliferative and proapoptotic action of Qu on tumor cell lines. In HepG2 cells, Qu causes cell-cycle arrest and apoptosis by inducing p53 phosphorylation and by stabilizing p53 both at the mRNA and protein level [131]. In HCT116 colon carcinoma cells, p53 contributes to Qu-mediated higher expression of NAG-1, which in turn triggers apoptosis [132]. It is interesting to note that the presence of p53 limits the effect of Qu, since when p53 is inhibited, cells become more sensitive to Qu-related cytotoxicity and Qu-related apoptosis. p53 elevates the p21 level, which may attenuate the proapoptotic effects of Qu in p53-wild-type tumor cells [126]. The H1299 lung carcinoma cell line, which is a p53-null cell line, is more susceptible to Qu-induced cytotoxicity than the A549 lung carcinoma cell line, which expresses a wild-type form of p53. In A549 cells, Qu-induced cytotoxicity and apoptosis are augmented when an inhibitor of p53 or an antisense oligonucleotide targeting p53 is used [126].

**Figure 3:**
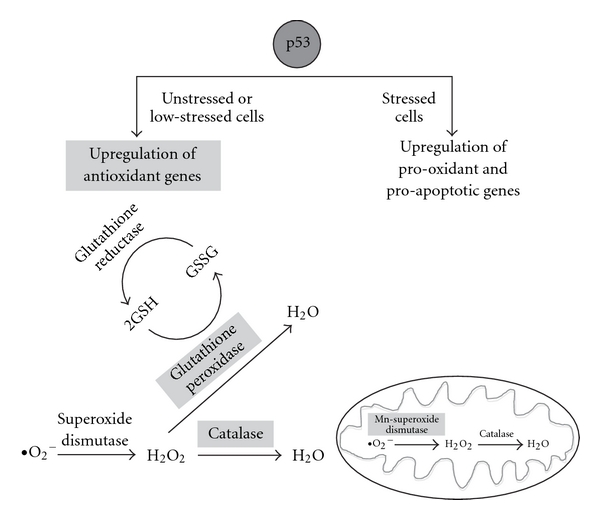
Effects of p53 in the intracellular oxidant system. According to a new model of p53-dependent regulation of ROS, p53 acts on the intracellular antioxidant system in both unstressed and low-stressed cells through the up-regulation of a series of genes (indicated in light gray boxes), including Gpx1, Mn-SOD2 and catalase. GPX catalyzes the oxidation of GSH into GSSG; Mn-SOD2 is localized in mitochondria and catalyzes the dismutation of superoxide (O_2_^−^) into oxygen and hydrogen peroxide (H_2_O_2_), whereas catalase catalyzes the decomposition of H_2_O_2_ to water and oxygen. When high levels of stress are present, p53 induces the transcription of several pro-oxidant as well as pro-apoptotic genes, such as bax (in dark gray circle), with the consequent release of cytochrome *c* from mitochondria, activation of caspases and induction of apoptosis.

The effect of the presence or absence of p53 on Qu-induced cytotoxicity and apoptosis is consistent with the involvement of Qu in the oxidative cell balance. This is well explained by a new model of the p53-dependent regulation of intracellular ROS (Figure 3). p53 can have an antioxidant function in unstressed or low-stressed cells through the regulation of a series of genes related to such activity. Among these genes, the most important are the microsomal GSH transferase homolog PIG12 [133], aldehyde dehydrogenase ALDH4A1 [134], Gpx1, Mn-superoxide dismutase SOD2 [135] and catalase [136]. Moreover, two members of the sestrin family, that is, SESN1 (pa26) and SESN2 (hi95), are also regulated by p53 [137, 138]. Sestrins act as components of the peroxiredoxin regeneration system in response to the massive bursts of H_2_O_2_ occurring during signal transduction. In unstressed cells, a good functionality of p53 is required for reducing intracellular ROS levels [139]. When p53 functions are blocked, or when its gene (TP53) is knocked out, a significant increase of intracellular ROS can be observed. ROS increase in p53-deficient cells is correlated with the down-regulation of the p53-regulated genes GPX1, SESN1 and SESN2, suggesting that p53 is required for maintaining these genes functional [139]. It is interesting to note that in a model of mild or severe H_2_O_2_-induced stress, cells knocked out by p53 exhibit much higher ROS levels than controls [140, 141]. Furthermore, p53 deficiency sensitizes cells to H_2_O_2_ damage, reducing viability and triggering apoptosis, and causes an excessive oxidation of DNA after challenge with H_2_O_2_ [140, 141].

### 4.6. Normal Cells Still Continue to Behave Normally

**Figure 4:**
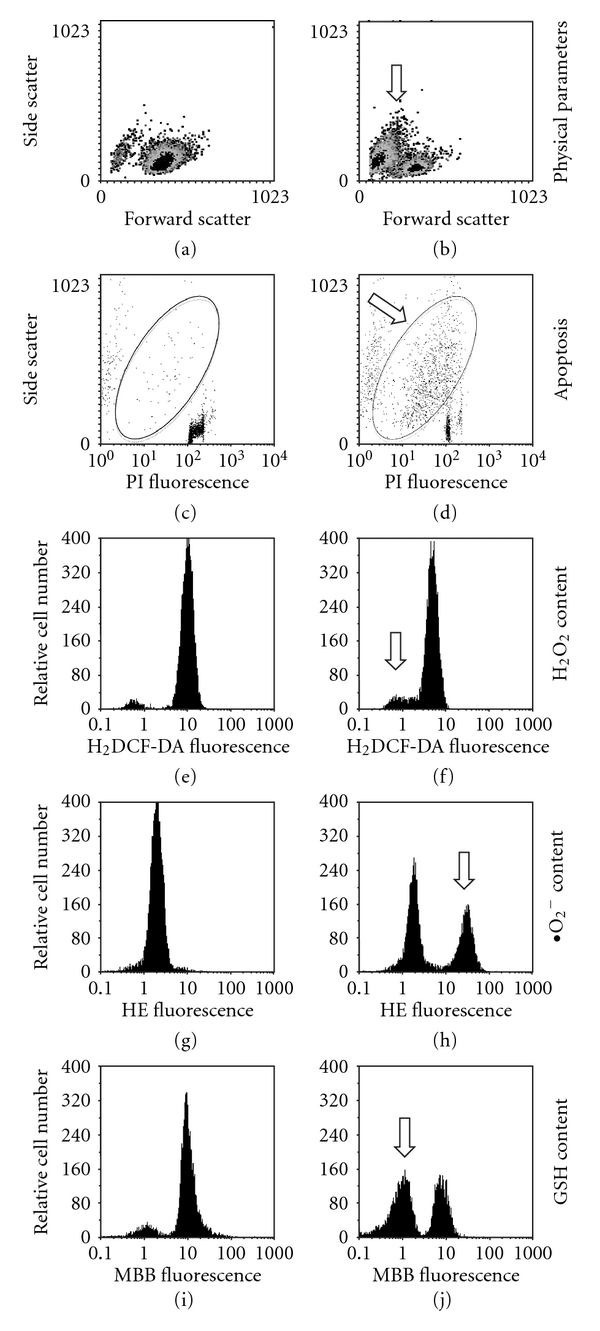
Changes in cell viability, apoptosis and content of H_2_O_2_, O_2_^−^ and GSH in U937 tumor cell line treated for 24 h with Qu 100 *μ*M. U937 cells treated with 100 *μ*M Qu were separately stained with four fluorescent dyes: propidium iodide (PI), 2′,7′-dichlorodihydrofluorescein diacetate (H_2_-DCFDA), hydroethidine (HE) and monobromobimane (MBB) for the quantification of nuclear DNA, intracellular H_2_O_2_, intracellular O_2_^−^ and intracellular GSH content, respectively. Cells were then analyzed by flow cytometry. In (a) and (b) the physical parameters (identified by forward and side scatter for cell dimension and granularity, resp.) of the cells under investigation are represented. Treating cells with 100 *μ*M Qu causes an increase in the number of cells with reduced forward scatter and increased side scatter, typical of apoptosis (indicated in b by an arrow). (c) and (d) Changes in DNA content (PI fluorescence) and side scatter in control and Qu-treated cells. In the ellipse, cells with hypodyploid DNA content and increased side scatter (i.e., those apoptotic) are present, and increase after treatment with Qu. (e) and (f) Show intracellular H_2_O_2_ content in both control and treated cells. Qu causes a shift to the left of the fluorescence peak (see, in (e), the histogram shift in relation to the fix position of the red bars) indicating a small reduction in H_2_O_2_ content, likely because of the concomitant increase in O_2_^−^. Qu also causes a small change in the percentage of cells that do not bind the dye, that is, those undergoing apoptosis (arrow), which are those evidenced in (d). (g) and (h) Represent intracellular O_2_^−^ content in the absence or in the presence of 100 *μ*M Qu. Treating cells with Qu significantly increases the amount of cells with high HE fluorescence (arrow), which represents an increase of intracellular O_2_^−^ content. (i) and (j) Show GSH content in the absence or after treatment with Qu 100 *μ*M. In the presence of Qu, MBB fluorescence decreases in a consistent number of cells (arrow), indicating that Qu is able to deplete intracellular GSH.

Despite the fact that the effects of Qu have been analyzed in a variety of cancer cells, little is known about its effects on normal, non-transformed human cells. In general, the most striking difference between normal and tumor cells is that tumor cell lines are prone to cell-cycle arrest and apoptosis at Qu concentrations that have no or little effect on non-transformed cells [142]. For example, in human lung embryonic fibroblasts and human umbilical vein endothelial cells, Qu exerts cytotoxicity by increasing intracellular ROS levels only when present at very high concentrations, that is, from 100 to 500 *μ*M [142]. Recently, we have shown that treating human peripheral blood lymphocytes (PBL) with Qu causes a loss of mitochondrial membrane potential only in a small amount of cells, and that this effect occurs only at high concentrations, that is, >100 *μ*M [101]. In activated or proliferating PBL, cell death seemed to be independent from the entrance into the cell cycle of resting lymphocytes (a phenomenon that typically occurs after antigenic stimulation). In this model, Qu did not even increase PBL susceptibility to CD95-induced apoptosis [101, 113]. It is important to note that the doses that were not effective for PBL (i.e., <50 *μ*M) were highly toxic for cancer cells or tumor cell lines (Figure 4).

Indeed, the ability of Qu to exert antiproliferative and proapoptotic effects on normal cells only at very high concentrations sharply contrasts with the low concentrations needed to exert the same effects on cancer cells that are, for example, 3.5 *μ*M for the B16-BL6 murine melanoma cell line [143], 25 *μ*M for PC-3 (p53-null cells) and DU-145 (p53-mutated cells) human prostate cancer cell lines [116] and 10 *μ*M for SK-Br3, MDA-MB-453 and MDA-MB-231 human breast carcinoma cells [114]. Interestingly, the proliferation of MCF-10A cells, which are normal breast epithelial cells, is not affected by 10 *μ*M of Qu [114] and, similarly, in the normal fibroblast cell line BG-9 Qu does not affect cell growth [116].

Qu protects mouse thymocytes from oxidative stress-mediated apoptosis [144]. The antioxidant activity of Qu was evaluated using the “G/GO system" in mouse normal thymocyte cells, in which hydroxyl radicals are constantly produced by glucose oxidase from glucose substrate. In this model, Qu pretreatment significantly reduced the G/GO-induced apoptosis of thymocytes and suppressed DNA-binding activity of redox state-sensitive transcription factors, such as NFkB, AP-1 and p53. The pretreatment of primary rat hippocampal cultures with Qu significantly decreased cytotoxicity and apoptosis induced by amyloid *β*-peptide [145]. Similarly, the effect of a conditioned medium obtained from astrocytes treated with the proinflammatory cytokine IL-1*β* and Qu has been evaluated on human neurons. In the presence of Qu, a significant decrease in neuronal apoptosis has been observed, which is caused by the inhibition of inflammatory mediators, including interleukin (IL)-6, IL-8 and monocyte chemoattractant protein 1, and the increase in the expression of superoxide dismutase and thioredoxin mediators [146].

In summary, what emerges from the few studies on healthy cells is that the concentrations of Qu that can be obtained with a diet rich in these flavonoids are capable of exerting significant effects on tumor cells, while not affecting cell cycle or cell activation of normal, non-transformed cells.

## 5. *In Vivo* Studies and Their Problems

### 5.1. Carcinogenesis in Different Animal Models

Although different aspects of the molecular mechanisms involved in the preventive effects of Qu on cancer have been covered, its efficacy *in vivo* as chemopreventer or chemoterapeutical has to be further elucidated. For this purpose, in recent years the effects of Qu have been studied in several animal models of carcinogenesis. Administration of Qu before the initiation stage of carcinogenesis reduced benzo(a)pyrene-induced lung tumor burden in mice which showed an increase in the activity of antioxidant enzymes, including superoxide dismutase, catalase, GSH peroxidase, GSH-S-transferase, GSH reductase and a decrease in the levels of lipid peroxides [147]. Similarly, administration of Qu supplements prior to exposure of azoxymethane as carcinogen drastically reduced the incidence of aberrant crypt *foci* and preneoplastic lesions in rat colon [148]. Qu was tested as a possible treatment for primary and invasive mammary carcinoma induced by dimethyl-benz-(*a*)-anthracene [149]. A direct injection of Qu into the tumor mass once a week for 4 weeks significantly reduced the volume of the neoplastic lesions.

In a recent study, treatment with both *N*-nitrosodiethylamine as cancer-inducer and Qu as preventer protected rats against hepatocarcinoma; this was accompanied by the maintenance of a correct intracellular oxidant/antioxidant status. In the presence of Qu, lipid peroxidation was inhibited, and GSH and GSH peroxidase exerted protective effects against oxidative damage [150]. When administered by intraperitoneal injection to mice previously engrafted with lung tumor cells, Qu had a growth-inhibitory activity [143]. Other than a significant dose-dependent delay in tumor growth, together with apigenin Qu displays a potent anti-invasive activity on the highly metastatic B16-BL6 melanoma cells *in vitro* [143]. Qu, in combination with resveratrol and catechins, was able to reduce distal metastatic invasions, especially to liver and bone, in nude mice through the up-regulation of the forkhead box O1 (FOXO1) and NFKBIA (IkappaBalpha) genes, which activate apoptosis and inhibit NF*κ*B activity [151].

### 5.2. The Problem of Solubility in Water and Possible Solutions

The limited solubility of Qu in water presents a major problem for its administration as a chemopreventer. Accordingly, many studies analyzed possible complexes able to transport Qu to various tissues [152, 153]. Promising studies have been obtained with polyethylene glycol (PEG) and sulfobutyl ether-7beta-cyclodextrin (SBE7betaCD) [154, 155]. The association of Qu to PEG (Q-PEGL) has been tested in different mouse models through intravenous injections. The biodistribution and the antitumor activity of Qu have been evaluated in mice bearing lung cancer and in mice bearing colon adenocarcinoma and hepatoma. Interestingly, Q-PEGL has a better solubility in water, and prolongs the circulation times of Qu in blood, enhancing its antitumor activity [155].

The use of Qu bound to SBE7betaCD carrier has been studied in the BDF1 mouse model of melanoma, after oral administration of such compounds. Qu-SBE7betaCD complex significantly improved anti-cancer activity of Qu, with decreased density of the microvessel within the melanoma [154].

Finally, another strategy to bypass the poor water-solubility of Qu was the use of a vesiculated form of Qu in galactosylated liposomes, able to bind galactosyl receptors on the surface of hepatic cells. In rats, Qu-galactosylated liposomes have been tested against diethyl nitrosamine induced hepatocarcinoma: a decrease in the number of both hyperplastic nodules and preneoplastic lesions in the rat liver have been reported [156].

## 6. Relevance for Human Health

The capacity of Qu to act as a chemotherapeutical compound is poorly studied, even if the combination of curcumin and Qu appears to reduce the number and size of ileal and rectal adenomas in patients with familial adenomatous polyposis, without the onset of an appreciable toxicity [157]. It is to note that all the epidemiological studies that often report contrasting data, do not have the possibility to evaluate the activity of Qu as such, but have to cope with the dietary intake of this flavonoid.

Qu can be considered a very interesting candidate for clinical applications in the prevention (or even in the treatment) of some forms of cancer, as several data consistently support its safety for human health and the lack of adverse effects, at least at the levels of the estimated dietary intake [158]. With particular regard to dietary supplementation, apparently controversial effects have been observed *in vitro* and *in vivo*. Indeed, although *in vitro* studies demonstrated the existence of a Qu-related mutagenicity, long-term *in vivo* toxicity studies failed to show any Qu-related promotion of carcinogenicity after oral administration.

Two mechanisms have been proposed to explain this dual aspect. First, Qu scarce absorption, together with almost complete metabolism in the intestinal tract, support the importance of Qu degradation for the elimination of its toxicity [159]. As a result of the first-pass effect, oral administration of Qu causes its almost complete metabolization, and metabolites still retain antioxidant properties [160]. Conversely, severe toxic effects can be exerted when Qu is administered intraperitoneally [161]. Second, multiple detoxifying mechanisms exist, *in vivo*, to limit the pro-oxidant effects of Qu. As far as human clinical trials are concerned, no significant adverse effects were reported after oral administration of Qu at doses up to 1000 mg day^−1^, corresponding to a high daily supplementation, for up to 12 weeks. Only one old study reported the presence of urinary bladder and intestinal tumors in rats fed with a dietary supplement of Qu of the order of 50 mg kg^−1^ body weight per day [162]. However, these results were not confirmed by any other long-term study performed with several-fold higher doses of Qu [163, 164], and in fact the occurrence of the aforementioned cancers was ascribed to the potential cross-contamination of the bracken fern diet with the carcinogen ptaquiloside [165].

Parallel to safety studies, exhaustive studies on efficacy of Qu in human beings are also needed. The sole clinical study performed with Qu has determined the serum concentration of a water-soluble pro-drug form of Qu, named QC12, after oral or intravenous administration to patients with different types of tumor, but did not evaluate any clinical effect [166]. It would be of extreme interest to expand this observation, and plan studies on different types of patients, ranging from those with treatable forms of cancer, to those who have failed any form of chemo- or radiotherapy.

At present, single natural chemicals are investigated in clinical trials to evaluate their potential chemopreventive activity on different types of cancer. For instance, lycopene and genistein are investigated for the prevention of prostate cancer, resveratrol and curcumin particularly for colon cancer, while green tea preferentially for solid tumors, lung and esophageal cancers [93]. The inclusion of Qu in this group will likely expand the possibility to fight against this or other types of human diseases. Finally, it is to note that the use of Qu is likely to be much higher than what we imagine, as it is freely sold almost everywhere, and easily available as dietary supplement (e.g., in the General Nutrition Corporation—GNC stores).

## 7. Conclusions

The studies of Qu on cellular models offer an almost exhaustive explanation of the mechanisms that link Qu to the oxidative cell balance and to the control of cell-cycle phases. Promising results have been obtained in the evaluation of the biological effects of Qu on both cancer and normal cells: the high toxicity of Qu for cancer cells, along with the characteristic to exert antiproliferative and proapoptotic effects on normal cells only at high concentrations are crucial aspects in the field of anticancer research, whose important goal is the identification of drugs that selectively kill tumor cells without damaging normal cells.

Results from cellular models invite major attention to study Qu in more complex and sophisticated animal models, such as those represented by animals with genetic defects in one or more genes that control oncogenesis, or with primary or secondary immune deficiencies. Furthermore, controlled clinical trials are needed to assess both the chemopreventive and chemoterapeutic effects of this molecule in a pure form. For this reason, investigations focused on pharmacokinetic and bioavailability in different regions of the organism are urgently needed.

## Funding

MIUR-PRIN 2008 (Project: “Regulation of Lon protease expression in response to oxidative damage to mitochondria" to A.C., partial).
